# Assessment of health-related quality of life and health status in patients with treatment-resistant depression treated with esketamine nasal spray plus an oral antidepressant

**DOI:** 10.1186/s12955-023-02113-1

**Published:** 2023-05-08

**Authors:** Carol Jamieson, Vanina Popova, Ella Daly, Kimberly Cooper, Wayne C. Drevets, Heather M. Rozjabek, Jaskaran Singh

**Affiliations:** 1 Janssen Research & Development, LLC, Milpitas, CA 95035 USA; 2 Janssen Research & Development, Beerse, BE Belgium; 3 Janssen Scientific Affairs, LLC, Titusville, NJ USA; 4 Janssen Research & Development, LLC, Spring House, PA USA; 5 Janssen Research & Development, LLC, San Diego, CA USA; 6 Janssen Research & Development, LLC, Raritan, NJ USA; 7 Present Address: Neurocrine Biosciences, San Diego, CA USA

**Keywords:** European Quality of Life Group, Five Dimension, Five Level (EQ-5D-5L), Esketamine, Health-related quality of life (HRQoL), Major depressive disorder, Sheehan Disability Scale, Treatment-resistant depression

## Abstract

**Background:**

Patients with treatment-resistant depression (TRD) report significant deficits in physical and mental health, as well as severely impaired health-related quality of life (HRQoL) and functioning. Esketamine effectively enhances the daily functioning in these patients while also improving their depressive symptoms. This study assessed HRQoL and health status of patients with TRD, who were treated with esketamine nasal spray and an oral antidepressant (ESK + AD) vs. placebo nasal spray and an AD (AD + PBO).

**Methods:**

Data from TRANSFORM-2, a phase 3, randomized, double-blind, short-term flexibly dosed study, were analyzed. Patients (aged 18–64 years) with TRD were included. The outcome assessments included the European Quality of Life Group, Five Dimension, Five Level (EQ-5D-5L), EQ-Visual Analogue Scale (EQ-VAS), and Sheehan Disability Scale (SDS). The health status index (HSI) was calculated using EQ-5D-5L scores.

**Results:**

The full analysis set included 223 patients (ESK + AD: 114; AD + PBO: 109; mean [SD] age: 45.7 [11.89]). At Day 28, a lower percentage of patients reported impairment in the ESK + AD vs. AD + PBO group in all five EQ-5D-5L dimensions: mobility (10.6% vs. 25.0%), self-care (13.5% vs. 32.0%), usual activities (51.9% vs. 72.0%), pain/discomfort (35.6% vs. 54.0%), and anxiety/depression (69.2% vs. 78.0%). The mean (SD) change from baseline in HSI at Day 28 was 0.310 (0.219) for ESK + AD and 0.235 (0.252) for AD + PBO, with a higher score reflecting better levels of health. The mean (SD) change from baseline in EQ-VAS score at Day 28 was greater in ESK + AD (31.1 [25.67]) vs. AD + PBO (22.1 [26.43]). The mean (SD) change in the SDS total score from baseline to Day 28 also favored ESK + AD (-13.6 [8.31]) vs. AD + PBO (-9.4 [8.43]).

**Conclusions:**

Greater improvements in HRQoL and health status were observed among patients with TRD treated with ESK + AD vs. AD + PBO.

**Trial registration:**

ClinicalTrials.gov Identifier: NCT02418585.

**Supplementary Information:**

The online version contains supplementary material available at 10.1186/s12955-023-02113-1.

## Background

Major depressive disorder (MDD) is a disabling illness with a lifetime prevalence globally of ~ 15% (> 300 million people worldwide) and 20.6% in US adults [1, 2]. Despite improved disease management and access to multiple classes of antidepressants (ADs), approximately 30% of patients with MDD do not achieve remission [3, 4]. Although there is no universal definition and a consensus has not yet been established, treatment-resistant depression (TRD) is commonly characterized as MDD with a lack of clinically meaningful improvement after treatment with at least 2 different oral antidepressant treatments taken at adequate doses for adequate duration (at least 6 weeks) in the current episode of depression [4–6].

TRD is associated with poor health-related quality of life (HRQoL) [7], higher unemployment [8], loss of productivity [7], and increased healthcare resource utilization and costs [9, 10]. Patients with TRD face more severe comorbidities [7, 11–13] and experience greater deterioration in their physical and mental health as well as increased suicidality [14–17] than patients with non-treatment resistant MDD. Symptoms of TRD interfere with daily activities and functioning, leading to a decline in work productivity and reduced job retention. Patients with TRD also use more healthcare resources, thus contributing to greater economic burden [7–10]. Consistent evaluation of diminished functional capacity and HRQoL is important in the management of patients with TRD in order to guide treatment in clinical settings [18]. The European Quality of Life, five Dimension, five Level (EQ-5D-5L) [19, 20] and the Sheehan Disability Scale (SDS) [21–23] are reliable and validated patient-reported outcome (PRO) measures for capturing and evaluating HRQoL and functional impairment of TRD from the patients’ perspective.

Recently, the N-methyl-D-aspartate receptor antagonist esketamine (ESK; the S-enantiomer of ketamine racemate) nasal spray, in conjunction with an oral AD, was approved by the U.S. Food and Drug Administration for the treatment of TRD and depressive symptoms in patients with MDD who have acute suicidal ideation or behavior [24]. ESK effectively improves depressive symptoms, daily functioning and HRQoL [25–28] and prevents relapse in TRD [29–31]. In the phase 3 long-term clinical trials, the treatment effects were sustained over a considerable period of time supporting its use as a long-term maintenance therapy [28]. Here we report the results of a secondary analysis of data from TRANSFORM-2 [32], a phase 3, randomized, double-blind, flexible-dose, active-controlled, multicenter study evaluating HRQoL and health status in patients with TRD who were treated with either ESK plus an oral AD (ESK + AD) or an oral AD plus placebo (AD + PBO).

## Methods

### Study design

Details of the study design and inclusion/exclusion criteria of TRANSFORM-2 have been previously published [32]. Briefly, the study consisted of 3 phases: (1) a 4-week screening and prospective observational phase; (2) a 4-week double-blind treatment phase; and (3) a posttreatment follow-up phase of up to 24 weeks. ESK 56 mg or 84 mg (or PBO) nasal spray was administered twice a week as a flexible dose for 4 weeks. All the patients received a newly initiated oral AD that was administered daily in an open-label manner throughout the 4-week treatment phase. Patients rated the impact of the study treatments on PRO measures (including EQ-5D-5L, SDS, etc.) prior to dosing at baseline and on Days 15 and 28 (Supplementary Figure 1). The study was conducted in accordance with the ethical principles of the Declaration of Helsinki International Conference on Harmonization, Good Clinical Practice guidelines, and applicable regulatory requirements. All patients provided written informed consent. Study protocols and amendments were approved by independent review board or ethics committee for each study site.

### Study assessments

Overall, health outcomes and socio-occupational disability were assessed using the EQ-5D-5L [19] and the SDS [22], respectively. The EQ-5D-5L consists of the EQ-5D descriptive system and the EQ-Visual Analogue Scale (EQ-VAS) [20]. The descriptive system comprises 5 dimensions (mobility, self-care, usual activities, pain/discomfort, and anxiety/depression) with each dimension scored on 5 levels (L1: no problems, L2: slight problems, L3: moderate problems, L4: severe problems, and L5: extreme problems). Patients were asked to select the most appropriate statement in each dimension that best matched their current health state “today”. Each dimension’s response was used to generate a health status index (HSI; anchored at 0 [health state value equal to dead] and 1 [full health]). Changes in the HSI on the order of 0.03 to 0.07 were considered the threshold for meaningful change for an individual patient [33, 34]. Individual scores from the 5 dimensions of the EQ-5D-5L were combined to obtain a 5L profile score or health state (e.g., a score of 1 for each dimension gives a 5L profile score of 11111). The Canadian value [35] set (time trade-off-based values set of the EQ-5D-5L for Canada) was used to obtain the weighted HSI values for all the countries participating in this study.

The EQ-VAS is designed to enable patients to quantify elements, which are relevant and important to each individual patient in determining their health status and may include concepts outside of the 5 dimensions of health in their overall health rating [20]. Patients self-rated their overall health status on a vertical visual analogue scale from 0 (‘The worst health you can imagine’) to 100 (‘The best health you can imagine’). Changes in the EQ-VAS on the order of 7 to 10 were recognized as a threshold for meaningful change for an individual patient [36].

The SDS is a brief, self-report tool that assesses functional impairment in 3 inter-related domains: work/school, social life, and family life [21–23]. It consists of a 5-item questionnaire. The first 3 items assess disruption in work/school, social life, and family life/home responsibilities. Patients rated the extent to which their symptoms were impaired in these 3 domains on a 10-point visual analog scale (0: not at all; 10: extremely). The 3 items were summed into a single dimensional measure (SDS total score) of global functional impairment that ranged from 0 to 30. A decrease in SDS total score from baseline indicates improvement. The last 2 items assess the days lost from school/work and days when underproductive, respectively (these 2 items are not included in the SDS total score). The recall period for this scale is 7 days. To assess response, the following thresholds were selected: scores ≤ 4 for each item and SDS total score ≤ 12 were considered a response, while scores ≤ 2 for each item and SDS total score ≤ 6 were considered a remission [37].

The changes in patient reported depressive symptoms were also evaluated, using the Patient Health Questionnaire – 9-Item (PHQ-9) scale. The 9 items represent the 9 symptom domains of the Diagnostic and Statistical Manual of Mental Disorders, Fifth Edition (DSM-5) MDD criteria and have been rated on a 4-point scale (0: not at all, 1: several days, 2: more than half the days, and 3: nearly every day). The sum of patient’s individual item responses represents the total score (range of 0 to 27), with severity defined as follows: 0–4 none/minimal symptoms, 5–9 mild symptoms, 10–14 moderate symptoms, 15–19 moderately severe and 20–27 severe symptoms. Higher total scores represent greater severity of depressive symptoms [38].

### Statistical analysis

Statistical analysis was performed on the full analysis set, which was defined as all randomized patients who received at least 1 dose of study medication (ESK or PBO nasal spray) and 1 dose of oral AD in the treatment phase. Descriptive statistics of actual values and changes from baseline by treatment group were provided for the weighted EQ-5D HSI, the EQ-VAS, and the individual EQ health dimensions at each time point for the double-blind treatment phase. Changes in the HSI and EQ-VAS scores from baseline to Day 28 were analyzed based on a mixed-effect model for repeated measures (MMRM) with treatment, day, country, class of oral AD (selective serotonin reuptake inhibitor [SSRI] or serotonin and norepinephrine reuptake inhibitor [SNRI]), and treatment-by-day as factors and baseline values as the covariates.

For the individual EQ health dimensions, the ESK + AD and AD + PBO groups were compared using a Cochran-Mantel–Haenszel Chi-square test adjusting for country and class of AD (SNRI or SSRI). Relative risk (RR) and 95% confidence interval (CI) were calculated. The change from baseline to Day 28 in SDS total score and PHQ-9 total score was evaluated using the same MMRM model as described above. Based on the pre-planned hierarchal testing of key secondary endpoints in the study, a serial gatekeeping (fixed sequence) approach was applied to adjust for multiplicity and to control type I error while evaluating changes in SDS total score, and PHQ-9 total score. Means (± standard error [SE]), mean changes (± SE) from baseline, and least squares (LS) mean changes (± SE) from baseline were presented graphically for the double-blind treatment phase for the observed cases and separately for the last observation carried forward (LOCF) evaluations.

## Results

### Study population

The full analysis set included 223 patients (ESK + AD: 114; AD + PBO: 109). Both treatment groups had similar demographic and baseline clinical characteristics (Supplementary Table 1). Most patients (87.9%) documented non-response to ≥ 2 antidepressant treatments at the start of the screening/prospective observational phase, with 55.2%, 20.6%, and 12.1% having 2, 3, and ≥ 4 antidepressant treatments with non-response, respectively. Subsequently after confirming non-response to the ongoing antidepressant, all patients were required to have non-response to ≥ 2 antidepressant treatments prior to randomization. Most of the patients (87.0%) had experienced ≥ 2 major depressive episodes (2–5: 71.3%; 6–10: 13.9%; > 10: 1.8%).

### EQ-5D-5L

**Fig. 1:**
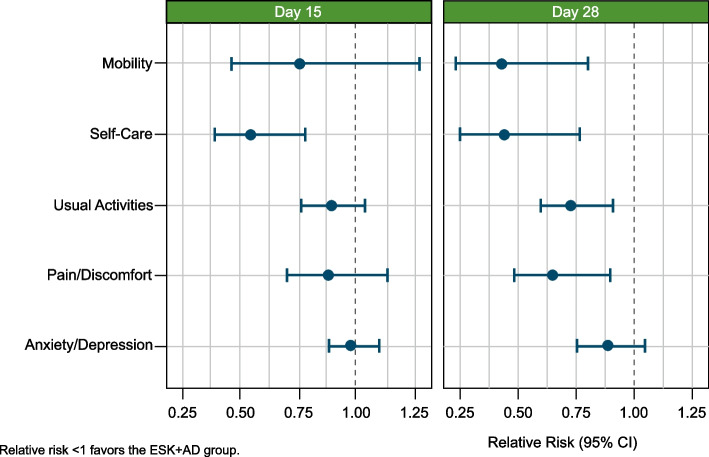
EQ-5D-5L individual dimensions – relative risk for problems. AD, antidepressant; CI, confidence interval; EQ-5D-5L, European Quality of Life-5 Dimension-5 Level; ESK, esketamine

The proportion of patients reporting impairment in each EQ-5D-5L dimension (grouped L2-L5 responses for each dimension) in the ESK + AD vs. AD + PBO groups at baseline, Day 15, and Day 28 and the percentage difference of patients reporting problems between the ESK + AD and AD + PBO groups are shown in Table 1. At Day 28, a lower percentage of patients reported impairment in the ESK + AD vs. AD + PBO group in all EQ-5D-5L dimensions: mobility (10.6% vs. 25.0%), self-care (13.5% vs. 32.0%), usual activities (51.9% vs. 72.0%), pain/discomfort (35.6% vs. 54.0%), and anxiety/depression (69.2% vs. 78.0%). A similar trend was observed at Day 15. Compared with the AD + PBO group, patients in the ESK + AD group were less likely to report impairments in self-care (RR = 0.549; 95% CI: 0.390, 0.774) at Day 15, and in mobility (RR = 0.428; 95% CI: 0.230, 0.797), self-care (RR = 0.436; 95% CI: 0.251, 0.756), usual activities (RR = 0.730; 95% CI: 0.587, 0.908), and pain/discomfort (RR = 0.659; 95% CI: 0.486, 0.893) at Day 28 (Fig. 1).

**﻿Table 1:** EQ-5D-5L individual dimensions – percentage of patients reporting problems in ESK + AD and AD + PBO groups

**Dimension**	**ESK + AD (%)**			**AD + PBO (%)**			**% difference (95% CI)**	
	**Baseline (*n* = 114)**	**Day 15 (*n* = 111)**	**Day 28 (*n* = 104)**	**Baseline (*n* = 109)**	**Day 15 (*n* = 104)**	**Day 28 (*n* = 100)**	**Day 15**	**Day 28**
**Mobility**	28.1	18.9	10.6	34.9	24.0	25.0	-5.1 (-16.1, 5.9)	-14.4 (-24.8, -4.1)
**Self-care**	53.5	27.0	13.5	53.2	50.0	32.0	-23.0 (-35.6, -10.3)	-18.5 (-29.8, -7.3)
**Usual activities**	94.8	71.2	51.9	92.7	79.8	72.0	-8.6 (-20.1, 2.8)	-20.1 (-33.1, -7.1)
**Pain/Discomfort**	68.5	52.3	35.6	74.3	59.6	54.0	-7.4 (-20.6, 5.9)	-18.4 (-31.8, -5.0)
**Anxiety/Depression**	98.3	84.7	69.2	99.9	86.5	78.0	-1.9 (-11.2, 7.5)	-8.8 (-20.8, 3.3)

*AD* antidepressant, *CI* confidence interval, *EQ-5D-5L* European Quality of Life-5 Dimension-5 Level, *ESK* esketamine, *PBO* placebo

**Fig. 2:**
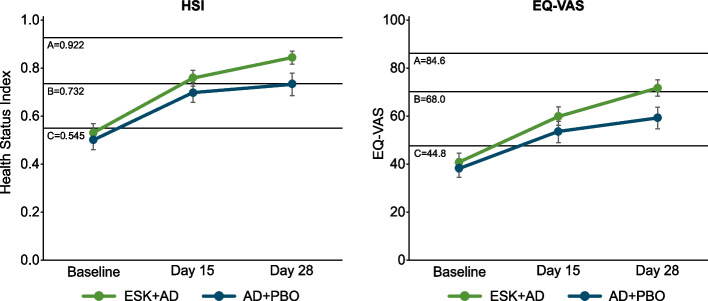
Mean (SE) HSI and EQ-VAS changes over time. AD + PBO, oral antidepressant plus placebo nasal spray; EQ-VAS, EuroQol Visual Analogue Scale; ESK + AD, esketamine nasal spray plus oral antidepressant; HSI, health status index; SE, standard error. For HSI; A: 18–29-year-old healthy adult; B: Depressive disorder; C: Senility without psychosis. The 3 horizontal lines indicate preference-based EQ-5D-5L index scores for a healthy 18–29-year-old individual, a patient with depressive disorder, and a senile patient without psychosis. These values have been added to visualize the changes observed in the current study and put them into clinical context. For EQ-VAS scores; Higher EQ-VAS scores indicate better health. A: general adult population in the US; B: patients with any cancer; C: patient with first episode or a new recurrent episode of depression. The 3 horizontal lines A, B, and C indicate EQ-VAS scores in the general adult population in the US, patients with any cancer, and a patient with first episode or a new recurrent episode of depression﻿

Mean HSI and EQ-VAS scores also improved relative to baseline during the 4-week double-blind treatment phase (Fig. 2). At Day 28, the mean (standard deviation [SD]) changes in the weighted HSI score relative to baseline was 0.310 (0.219) for the ESK + AD group and 0.235 (0.252) for the AD + PBO group, with higher scores reflecting better levels of health (Fig. 2). Based on the MMRM model, the LS mean (95% CI) treatment difference between the groups was 0.085 (0.036, 0.133). At Day 28, the mean (SD) changes in the EQ-VAS score relative to the baseline was 31.1 (25.7) for the ESK + AD group and 22.1 (26.4) for the AD + PBO group (Fig. 2). Based on the MMRM model, the LS mean (95% CI) treatment difference between the groups was 10.9 (5.22, 16.48).

### SDS

**Fig. 3:**
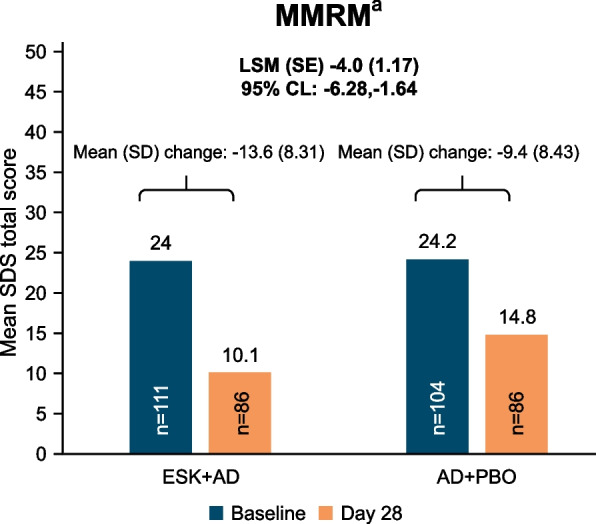
Change in the SDS score among patients included in the study using MMRM. AD + PBO, oral antidepressant plus placebo nasal spray; CI, confidence interval; ESK + AD, esketamine nasal spray plus oral antidepressant; LSM, least square mean; MMRM, mixed-effects model using repeated measures; SDS, Sheehan Disability Scale; SE, standard error. ^a^Test for treatment effect is based on mixed model for repeated measures (MMRM) with change from baseline as the response variable and the fixed effect model terms for treatment), day, country, class of oral antidepressant (SNRI or SSRI), and treatment-by-day and baseline value as a covariate. A negative difference favors the ESK + AD group. Note: SDS total score ranges from 0 to 30; a higher score indicates greater impairment. Negative change in score indicates improvement

Results for the change in SDS total score numerically favored treatment with ESK + AD over AD + PBO. The mean (SD) change from baseline to Day 28 was -13.6 (8.31) for the ESK + AD group and -9.4 (8.43) for the AD + PBO group. Based on the MMRM model, the LS mean difference (95% CI) between the ESK + AD group and the AD + PBO group was -4.0 (-6.28, -1.64) (Fig. 3).

A responder analysis based on SDS total score at Day 28 found that 49 of 86 (57.0%) patients in the ESK + AD group and 34 of 86 (39.5%) patients in the AD + PBO group were responders, while 34 of 86 (39.5%) patients in the ESK + AD group and 18 of 86 (20.9%) patients in the AD + PBO group were in remission.

### PHQ-9 total score

The mean change from baseline (SD) to Day 28 in the PHQ-9 total score favored ESK + AD group (-13.0 [6.42]) over AD + PBO group (-10.2 [7.80]; group difference: LS mean [95%CI] = -2.4 [-4.18; -0.69]). The change in PHQ-9 total score from baseline to the endpoint of the double-blind induction phase (LOCF) were consistent with the MMRM analysis (ESK + AD: -12.2 [6.87] vs. AD + PBO: -10.1 [7.87]; group difference: LS mean [95%CI] = -2.2 [-3.93; -0.40]).

Improvement in depression severity from baseline to Day 28, favored treatment with ESK + AD over AD + PBO.

Examination of severity levels of depression at Day 28 by treatment group showed none/minimal (PHQ-9 total score < 5; ESK + AD: 37 [35.6%] vs AD + PBO: 31 [31.0%]), mild (total score 5–9; ESK + AD: 38 [36.5%] vs. AD + PBO: 29 [29.0%]), moderate (total score 10–14) ESK + AD: 16 [15.4%] vs. AD + PBO: 9 [9.0%]), moderately severe (total score 15–19) ESK + AD: 7 [6.7%] vs. AD + PBO: 13 [13.0%]), and severe (total score ≥ 20; ESK + AD: 6 [5.8%], AD + PBO: 18 [18.0%]). The data showed that the AD + PBO group had a higher proportion of patients with severe depression and the ESK + AD group had a lower proportion of patients with none/mild/moderate depression.

## Discussion

The results of this secondary analysis of data from TRANSFORM-2 evaluating HRQoL and health status in patients with TRD demonstrated clinically meaningful responses with ESK + AD vs. AD + PBO using the EQ-5D-5L, PHQ-9, and the SDS scores. The proportion of patients who reported impairment in each of the 5 EQ-5D-5L health state dimensions decreased from baseline to the end of the 4-week double-blind treatment phase in both treatment groups. Results in each of the 5 dimensions of EQ-5D-5L, the weighted HSI score (based on responses in all 5 dimensions), and the EQ-VAS score numerically favored the ESK + AD group over the AD + PBO group, and the 95% CI for the mean difference between groups at Day 28 did not overlap 0 in 4 of the 5 dimensions. Notably the category that showed the greatest number of patients reporting problems at Day 28 was for the depression/anxiety dimension. This may be explained by the double-barreled question combining anxiety and depression where patients may have responded based on either symptom. Additionally, while patients in both treatment groups showed clinically meaningful improvement in depression symptoms according to Montgomery Asberg Depression Rating Scale (MADRS) scores and PHQ-9 scores, not all patients achieved complete resolution of depression and still reported symptoms of mild depression. Responses for level 2 symptoms (I am slightly anxious or depressed) were 41.3% for ESK + AD and 29% for ESK + PBO and support this assessment. These factors may explain the high level of patients reporting problems in the depression/anxiety EQ-5D-5L dimension.

At the end of the double-blind treatment phase, patients treated with ESK + AD self-reported greater improvements from baseline in functioning and associated disability as assessed by the SDS scores than those treated with AD + PBO. Importantly, consistent advantages in patient reported HRQoL and functioning improvement were observed in this secondary analysis, supporting the clinical meaningfulness of the improvement observed in the primary endpoint^25^ from the patients’ perspective. Self-reported severity of depression measured by the PHQ-9 showed improvement in this study, with decrease in total score from baseline to the end of the 4-week double-blind period. Response to treatment measured by the severity level of depression symptoms on the PHQ-9 was numerically greater in the esketamine treatment group as demonstrated by percentage of patients at none/minimal and mild severity level. This is also supported by data showing increased responder rates, determined by within-patient meaningful change threshold, for patients treated with ESK + AD [39].

Data collected on HRQoL and health status using the EQ-5D-5L provide additional context for the improvement in depressive symptoms noted in the results from the primary efficacy analysis [25]. Some of these health state dimensions (e.g., mobility, self-care, usual activities) may not be considered in traditional assessments of treatment outcome in depression. The weighted HSI reflects how good or bad a health state is according to the preferences of the general population; while the EQ-VAS can capture problems or considerations that are not assessed within the 5 dimensions of the EQ-5D-5L, potentially revealing additional information relevant to patients with TRD. Consistent with the previously published data [40–42], patients with TRD who were included in the TRANSFORM-2 study, also presented with diminished functionality and HRQoL during enrollment into the study. However, the increase in HSI scores and decrease in SDS total score from baseline, established the efficacy of ESK + AD. Two published studies provided mean EQ-5D index scores for patients with depression in the US and UK [43, 44]. Notably, in TRANSFORM-2, the mean HSI scores at baseline in both treatment groups were considerably lower (suggesting worse health status) than those reported for patients with depression in these published studies. However, both treatment groups improved after treatment during the double-blind treatment phase, with greater improvement observed in those treated with ESK + AD. At the end of the double-blind treatment phase, the mean HSI scores either resembled or were higher than those reported in the published studies, although these comparisons should be interpreted with caution given the different data sources for the index score values.

While EQ-5D-5L is a generic measure of quality of life across 5 dimensions, the SDS assesses functional impairment and associated disability across 3 domains (work/school, social life/leisure activities, and family life/home responsibilities). As improvement in function is often distal to the improvement in depressive symptoms for patients with TRD, it is notable that patients treated for 4 weeks with ESK + AD reported greater improvement in function than those treated with AD + PBO. Furthermore, a combination of EQ-5D-5L and SDS supports the overall impact of the disease and treatment on HRQoL.

The improvement in HRQoL with ESK + AD may translate into enhancement of the patients’ overall ability to perform daily activities, concentrate on work, and engage more with family/friends, as demonstrated in an analysis of responses to semi-structured interviews conducted in patients who received long-term esketamine treatment [26]. The results of the current analysis stress the importance of including HRQoL measures as a routine assessment in patients with TRD irrespective of the treatment they receive. Identifying problems early may help in resolving them and preventing further complications. Moreover, improving patients’ perceptions of their disease and effect of the prescribed treatment may also enhance patient engagement in TRD management.

The results of this study must be viewed with caution given some notable limitations. Esketamine treatment has known transient dissociative effects that are difficult to blind and could have biased patient responses [24, 45]. Since this analysis used self-reported PRO data, strategies to reinforce blinding in the original study such as clinician-rated assessments performed by blinded independent raters, could not be utilized. Consequently, potential bias in self-reported responses to PROs due to an individual’s perception of treatment assignment cannot be ruled out. In addition, as this was a flexible dose study, dose–response relationships could not be examined since direct comparisons between dosage groups could not be conducted. Another limitation of this analysis is that the patient-reported changes in functional impairment and associated disability measured by the SDS and patient-reported changes in their depressive symptoms measured by PHQ-9, could not be formally evaluated statistically as the onset of clinical response at Day 2 (24 h) on the primary outcome measure (change from baseline on the MADRS) was not statistically significant (pre-specified key secondary endpoint in the study protocol). However, results from the SDS assessments were consistent with the results from the primary efficacy analysis. PHQ-9 results confirm benefits of the therapy in patients assessed depressive symptoms as observed in other clinical studies [39, 46].

## Conclusions

Significant improvement in HRQoL, health status, and functional outcomes was observed among patients with TRD treated with ESK + AD compared with those treated with AD + PBO within 4 weeks of initiating treatment. ESK in combination with an oral AD appears to offer meaningful benefit as a treatment option for patients with TRD who generally have substantial HRQoL and functioning limitations as a result of their depressive illness.

## Supplementary Information

**Additional file 1: Supplementary Table 1.** Baseline characteristics. **Supplementary Figure 1.** Study design.

## Data Availability

All data generated or analyzed during this study are included in this published article. The data sharing policy of Janssen Pharmaceutical Companies of Johnson & Johnson is available at https://www.janssen.com/clinical-trials/transparency. As noted on this site, requests for access to the study data can be submitted through Yale Open Data Access [YODA] Project site at http://yoda.yale.edu.

## Abbreviations

AD: Antidepressant
CI: Confidence interval
DSM-5: The Diagnostic and Statistical Manual of Mental Disorders, Fifth Edition
EQ: European quality
EQ-5D-5L: European Quality of Life Group, 5 Dimension, 5 Level
EQ-VAS: European Quality Visual Analogue Scale
ESK: Esketamine
HRQoL: Health-related quality of life
HSI: Health Status Index
LOCF: Last observation carried forward
LS: Least square
MDD: Major depressive disorder
MMRM: Mixed-effect model for repeated measures
PBO: Placebo
PHQ-9: Patient Health Questionnaire – 9 Item
PRO: Patient-reported outcome
RR: Relative risk
SD: Standard deviation
SDS: Sheehan Disability Scale
SE: Standard error
SNRI: Serotonin and norepinephrine reuptake inhibitor
SSRI: Selective serotonin reuptake inhibitor
TRD: Treatment-resistant depression
UK: United Kingdom
US: United States
