# Supplementary material for: Assessment of health-related quality of life and health status in patients with treatment-resistant depression treated with esketamine nasal spray plus an oral antidepressant
Source: Health Qual Life Outcomes. 2023 May 8;21:40. doi: 10.1186/s12955-023-02113-1 (PMC10169482; doi:10.1186/s12955-023-02113-1)
Supplement: Supplementary file 1 — Additional file 1: Supplementary Table 1. Baseline characteristics. Supplementary Figure 1. Study design. [file 12955_2023_2113_MOESM1_ESM.docx]

**Supplementary File**

**Assessment of Health-Related Quality of Life and Health Status in Patients with Treatment-Resistant Depression Treated with Esketamine Nasal Spray plus an Oral Antidepressant**

Carol Jamieson^1^*, Vanina Popova^2^, Ella Daly^3^, Kimberly Cooper^4^, Wayne C. Drevets^5^, Heather M. Rozjabek^6^, Jaskaran Singh^4,7^

^1^Janssen Research & Development, LLC, Milpitas, CA, USA

^2^Janssen Research & Development, Beerse, BE, Belgium

^3^Janssen Scientific Affairs, LLC, Titusville, NJ, USA

^4^Janssen Research & Development, LLC, Spring House, PA, USA

^5^Janssen Research & Development, LLC, San Diego, CA, USA

^6^Janssen Research & Development, LLC Raritan, NJ, USA

^7^ Present Address: Neurocrine Biosciences, San Diego, CA, USA

***Corresponding Author:**

Carol Jamieson, BSc, FIBMS

Janssen Research & Development, LLC

Milpitas, CA 95035

Tel: (650) 804 0224

Email: [cjamies2@its.jnj.com](mailto:cjamies2@its.jnj.com)

**Supplementary Table 1: Baseline characteristics**

|  | **ESK+AD (n=114)** | **AD+PBO (n=109)** | **Total (N=223)** |
| --- | --- | --- | --- |
| Age, years (mean [SD]) | 44.9 (12.58) | 46.4 (11.14) | 45.7 (11.89) |
| Age groups, n (%) |  |  |  |
| 18-44 years | 54 (47.4) | 40 (36.7) | 94 (42.2) |
| 45-64 years | 60 (52.6) | 69 (63.3) | 129 (57.8) |
| Sex, n (%) |  |  |  |
| Women | 75 (65.8) | 63 (57.8) | 138 (61.9) |
| Race, n (%) |  |  |  |
| White | 106 (93.0) | 102 (93.6) | 208 (93.3) |
| Black/African American | 6 (5.3) | 5 (4.6) | 11 (4.9) |
| Asian | 1 (0.9) | 1 (0.9) | 2 (0.9) |
| Multiple | 1 (0.9) | 1 (0.9) | 2 (0.9) |
| Weight, kg (mean [SD]) | 79.30 (20.140) | 82.67 (19.468) | 80.95 (19.842) |
| Height, cm (mean [SD]) | 169.23 (10.179) | 169.81 (9.953) | 169.51 (10.051) |
| BMI, kg/m^2^ (mean [SD]) | 27.5 (5.84) | 28.6 (6.24) | 28.1 (6.05) |
| BMI category, kg/m^2^ (n [%]) |  |  |  |
| Underweight<18.5 | 1 (0.9) | 2 (1.8) | 3 (1.3) |
| Normal 18.5-<25 | 41 (36.0) | 28 (25.7) | 69 (30.9) |
| Overweight 25-<30 | 41 (36.0) | 36 (33.0) | 77 (34.5) |
| Obese 30-<40 | 28 (24.6) | 39 (35.8) | 67 (30.0) |
| Morbidly obese ≥40 | 3 (2.6) | 4 (3.7) | 7 (3.1) |
| Employment status^*^, n (%) |  |  |  |
| Employed | 68 (59.6) | 63 (57.8) | 131 (58.7) |
| Unemployed | 34 (29.8) | 35 (32.1) | 69 (30.9) |
| Other | 12 (10.5) | 11 (10.1) | 23 (10.3) |
| Hypertension^**^, n (%) | 18 (15.8) | 27 (24.8) | 45 (20.2) |
| Country, n (%) |  |  |  |
| Czech Republic | 30 (26.3) | 28 (25.7) | 58 (26.0) |
| Germany | 10 (8.8) | 10 (9.2) | 20 (9.0) |
| Poland | 20 (17.5) | 18 (16.5) | 38 (17.0) |
| Spain | 9 (7.9) | 9 (8.3) | 18 (8.1) |
| United States | 45 (39.5) | 44 (40.4) | 89 (39.9) |
| Region, n (%) |  |  |  |
| Europe | 69 (60.5) | 65 (59.6) | 134 (60.1) |
| North America | 45 (39.5) | 44 (40.4) | 89 (39.9) |
| Class of oral antidepressant, n (%) |  |  |  |
| SNRI | 77 (67.5) | 75 (68.8) | 152 (68.2) |
| SSRI | 37 (32.5) | 34 (31.2) | 71 (31.8) |
| Oral Antidepressants, n (%) |  |  |  |
| Duloxetine | 60 (52.6) | 61 (56.0) | 121 (54.3) |
| Escitalopram | 21 (18.4) | 17 (15.6) | 38 (17.0) |
| Sertraline | 16 (14.0) | 16 (14.7) | 32 (14.3) |
| Venlafaxine extended release (XR) | 17 (14.9) | 15 (13.8) | 32 (14.3) |
| Psychiatric History |  |  |  |
| Age at MDD diagnosis (years) |  |  |  |
| Mean (SD) | 32.1 (12.53) | 35.3 (13.04) | 33.7 (12.86) |
| No. of previous antidepressant medications^***^, n (%) |  |  |  |
| 1 | 9 (7.9) | 18 (16.5) | 27 (12.1) |
| 2 | 69 (60.5) | 54 (49.5) | 123 (55.2) |
| 3 | 24 (21.1) | 22 (20.2) | 46 (20.6) |
| 4 | 7 (6.1) | 13 (11.9) | 20 (9.0) |
| 5 | 3 (2.6) | 1 (0.9) | 4 (1.8) |
| 6 | 1 (0.9) | 1 (0.9) | 2 (0.9) |
| 9 | 1 (0.9) | 0 | 1 (0.4) |
| No. of major depressive episodes including current episode, n (%) | | | |
| 1 | 15 (13.2) | 14 (12.8) | 29 (13.0) |
| 2-5 | 81 (71.1) | 78 (71.6) | 159 (71.3) |
| 6-10 | 16 (14.0) | 15 (13.8) | 31 (13.9) |
| >10 | 2 (1.8) | 2 (1.8) | 4 (1.8%) |
| Family history, n (%) |  |  |  |
| Depression | 51 (44.7) | 56 (51.4) | 107 (48.0) |
| Anxiety Disorder | 10 (8.8) | 16 (14.7) | 26 (11.7) |
| Bipolar Disorder | 8 (7.0) | 11 (10.1) | 19 (8.5) |
| Schizophrenia, | 6 (5.3) | 4 (3.7) | 10 (4.5) |
| Alcohol Abuse | 18 (15.8) | 20 (18.3) | 38 (17.0) |
| Substance Abuse | 8 (7.0) | 4 (3.7) | 12 (5.4) |

AD, antidepressant; BMI, body mass index; ESK, esketamine; n, number of patients; PBO, placebo; SD, standard deviation; SNRI, serotonin and norepinephrine reuptake inhibitors; SSRI, selective serotonin reuptake inhibitors.
*Any type of employment includes: any category containing “Employed”, sheltered work, housewife or dependent husband, and student; any type of unemployment includes: any category containing “Unemployed”; Other include retired and no information available.
**Hypertension status is classified as Yes if hypertension is recorded in medical history.
*** Number of antidepressant medications with non-response (defined as <=25% improvement) taken for at least 6 weeks during the current episode as obtained from the MGH-ATRQ, Massachusetts General Hospital – Antidepressant Treatment Response Questionnaire.

**Supplementary Figure 1: Study design**


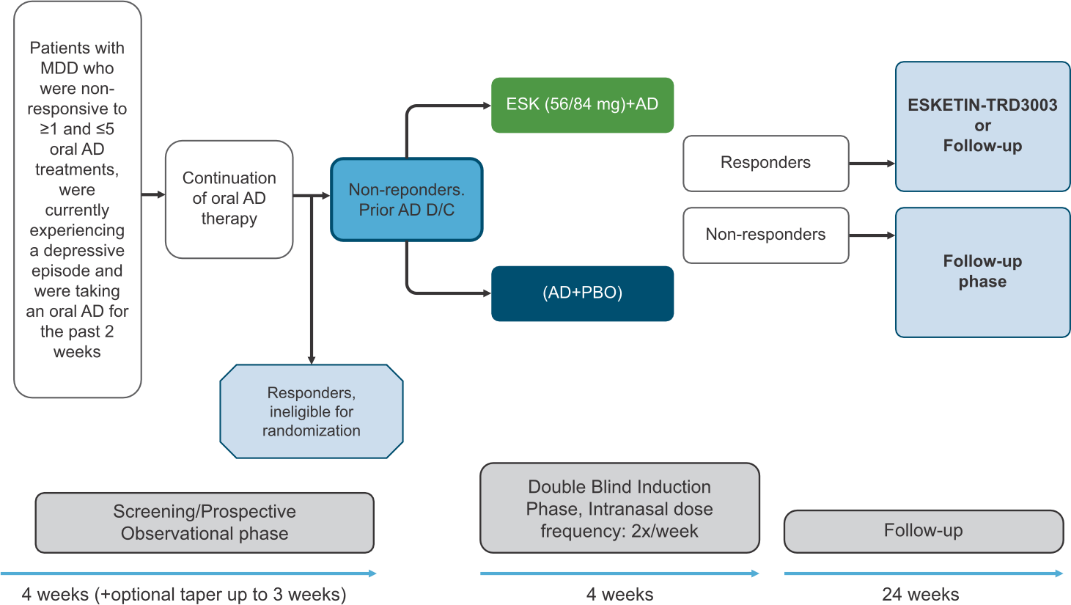


AD, antidepressants; D/C, discontinuation; ESK, esketamine; MDD, major depressive disorder; PBO, placebo.
